# PF127 hydrogel-assisted in-situ *Staphylococcus aureus* biofilm fabrication endowing pathological animal model for chronic osteomyelitis

**DOI:** 10.3389/fmicb.2026.1914727

**Published:** 2026-10-05

**Authors:** Jiawen Wang, Fuchao Chu, Qing Zhang, Xiangyu Qi, Liudong Zhang, Xu Zhang, Yazhong Zhang, Yongxiang Lv, Wenbo Li, Na Cai, Dekun Zhang, Ziqiang Zhu

**Affiliations:** 1 Department of Orthopaedics, The Second Affiliated Hospital of XuZhou Medical University, Xuzhou, China; 2 Graduate School of Xuzhou Medical University, Xuzhou, Jiangsu, China; 3 China University of Mining and Technology, Xuzhou, Jiangsu, China

**Keywords:** animal model, bacterial adhesion, biofilm, chronic osteomyelitis, hydrogel, infection

## Abstract

Chronic osteomyelitis driven by biofilms remains difficult to treat and thus the exploration of its clinical therapy and pathological mechanism largely depend on effective animal models. However, standardized and efficient methods for establishing stable and reproducible animal models for chronic osteomyelitis are still lacking. This study developed a Hydrogel Assisted In-situ Biofilm Fabrication (HAIBF) method by coupling thermosensitive Pluronic F-127 (PF127) hydrogel with biofilm fragments, which stably mimic the pathological process of biofilm-infected chronic osteomyelitis. Forty-eight rats were randomly allocated into four groups: a planktonic bacteria injection group, a direct biofilm injection group, a PF127-loaded biofilm injection group, and a blank control group. Rats were euthanized, and tibial samples were harvested once a week for the following month after infection. Macroscopic examination, SEM, CLSM, micro-CT with three-dimensional reconstruction and quantitative analysis, and H&E staining were combined to evaluate biofilm localization, bone destruction, and tissue pathology. PF127 facilitated the stable localization of biofilm within bone tissue. Collectively, these results validated the successful establishment of a chronic osteomyelitis model characterized by persistent biofilm infection, pathological bone remodeling, and impaired bone repair. HAIBF integrates low-metabolism biofilm fragments with an injectable, thermoresponsive PF127 hydrogel to establish an implant-free bone infection model that undergoes *in situ* gelation and promotes the local retention of biofilm-derived material. Within a relatively short infection progression period, this model reliably reproduces persistent biofilm-associated infection and selected pathological features of chronic osteomyelitis, thereby providing a more stable and consistent experimental platform for mechanistic studies and therapeutic evaluation.

## 1 Introduction

Chronic osteomyelitis (CO) is a refractory bone infection driven by biofilms (Pincher et al., 2019; Wang S. et al., 2023), characterized by a high recurrence rate (up to 33%) and substantial treatment costs (Camilleri-Brennan et al., 2023; Huang et al., 2016; Pimentel de Araujo et al., 2021; Ren et al., 2023). Developing reproducible animal models that precisely recapitulate its chronic and relapsing pathological process is therefore crucial for advancing novel therapeutic strategies (Billings and Anderson, 2022; Debs et al., 2024).

Currently, the model establishment of chronic osteomyelitis often employs the suspension bacteria injection method, which is simple to operate but suffers from issues such as unstable chronic infection characteristics and non-uniform biofilm formation (Li and Lai, 2024; Percie du Sert et al., 2020). Therefore, To better recapitulate clinically relevant, biofilm-associated chronic infections, several studies have proposed alternative model establishment strategies involving biofilm-laden implants (Meroni et al., 2022). These models simulate the presence of internal fixation devices, bacterial adhesion and biofilm formation, bone–implant interface responses, infection-related impairment of bone healing, and fixation failure, while also enabling the evaluation of clinically relevant therapeutic strategies, including antimicrobial materials, local drug delivery, and implant retention or removal (Huang et al., 2023). However, implant properties and handling can substantially alter infection, bone healing, and treatment responses, confounding model interpretation. Consequently, findings may have limited cross-species and clinical generalizability, particularly in short-term or nonphysiological models. Additionally, because the size of the exogenously introduced biofilm cannot be controlled, the excessively high local bacterial load may easily induce acute infection (Roux et al., 2021). As a result, to overcome the limitations of existing establishment of the infection model methods, we have established a new incorporation method of biofilm in order to enhance the reliability and reproducibility of the chronic osteomyelitis model.

To simulate the pathological process of biofilm-associated infections, this study employed a thermosensitive PF127 hydrogel as a coupling agent to interconnect fragmented biofilms, thereby facilitating *in situ* generation of a chronic osteomyelitis biofilm model in vivo, named as Hydrogel Assisted In-situ Biofilm Fabrication (HAIBF). Leveraging the softness and biocompatibility of PF127, the system allows for sustained release and protection of the interconnected biofilm fragments (Eltaher et al., 2022; Sayed et al., 2023; Se et al., 2025; Tang et al., 2023; Yang et al., 2025). This approach not only effectively circumvents acute infection and premature biofilm clearance but also supports the establishment of a persistent and stable microenvironmental colonization at the infection site. Furthermore, compared with conventional establishment of the infection model methods, HAIBF enables earlier and more reliable recapitulation of key pathological hallmarks of chronic osteomyelitis, thereby offering a robust and efficient platform for preclinical screening of relevant therapeutic strategies.

## 2 Materials and methods

### 2.1 Determination of the gelation temperature of PF127 thermogel

The gelation temperature of PF127 (Sigma-Aldrich,USA;NO.2443) solution was determined using the test tube inversion method. Ten PF127 aqueous solutions with concentrations ranging from 10 to 20 wt% were prepared by stirring at room temperature (25°C) until complete dissolution, yielding clear, bubble-free solutions. Two milliliters of each solution was transferred into glass vials and placed in a programmable temperature-controlled water bath. Starting at 25°C, the temperature was increased at 1°C intervals up to 45°C, with a 3-minute equilibration period at each step. The vials were then inverted to observe fluidity. The lowest temperature at which the solution ceased to flow (i.e., formed a gel) was recorded as the gelation temperature. The gel-sol transition upon cooling was subsequently observed using the same temperature intervals (Peng et al., 2022; Russo and Villa, 2019).

### 2.2 PF127 concentration screening and rheological characterization

Based on the results from section “2.1 Determination of the gelation temperature of PF127 thermogel”, three PF127 concentrations (10%, 15%, and 20%) were selected for rheological testing. Oscillatory temperature sweep tests were performed using a rotational rheometer (DHR-3, TA Instruments, New Castle, DE, USA). A parallel-plate geometry (40 mm diameter, 0.5 mm gap) was used with a fixed frequency of 1 Hz and a strain of 1%. The temperature was ramped from 25 to 45 °C at a rate of 0.5°C/min. The storage modulus (G′) and loss modulus (G″) were recorded as functions of temperature, and the results were plotted on a logarithmic scale (Qing et al., 2023).

### 2.3 Determination of PF127 degradation rate *in vivo*

Male Sprague-Dawley (SD) rats (250–300 g; Nanjing Laboratory Animal Technology Co., Ltd., Nanjing, Jiangsu, China) were anesthetized via intraperitoneal injection of 1% sodium pentobarbital (30 mg/kg;Sigma-Aldrich,USA;NO.1175). The dorsal fur was shaved and disinfected. A 2-cm incision was made on each side of the midline, and a subcutaneous pocket was created via blunt dissection. A pre-cooled syringe was used to inject 500 μL of 15% PF127 solution into each pocket, where it formed a gel upon reaching body temperature. The incision was closed using simple interrupted sutures with 4-0 absorbable polyglactin 910 sutures. The remaining gel was examined at 24, 48, 72, 96, 120, and 144 h post-implantation (*n* = 3 per time point) (Lin et al., 2024; Liu et al., 2025).

### 2.4 Effect of PF127 on bacterial growth by disk diffusion assay

*S. aureus* ATCC 25923 was selected as a well-characterized reference strain to standardize bacterial and biofilm preparation during the initial development of the HAIBF model. Its biofilm-forming capacity was evaluated using a crystal violet microtiter plate assay before the *in vivo* experiments. Mueller-Hinton agar plates were inoculated with a bacterial suspension adjusted to a 0.5 McFarland standard (∼1.5 × 10^8^ CFU/mL). Sterile blank paper disks were placed on the agar surface. Ten microliters of each test solution was applied to individual disks: 10% PF127 solution, 25% PF127 solution (representing extreme concentrations within the tested range), physiological saline (negative control), and penicillin solution (positive control). Plates were inverted and incubated at 37 °C for 18 h, after which the diameter of the inhibition zone (including the disk) was measured using a caliper.

### 2.5 Cell viability staining (live/dead assay)

PF127 powder was dissolved in pre-cooled sterile PBS (MCE;USA,NO.P750064) at 4°C with gentle stirring until a homogeneous solution was obtained. The solution was sterilized by filtration through a sterile 0.22-μm membrane filter rather than by autoclaving, because exposure to high temperature may affect the physicochemical and thermoresponsive gelation properties of PF127 (Beard et al., 2021). The sterilized solution was stored at 4°C. Two hundred microliters of sterile 15% PF127 solution was pipetted into wells of a 12-well plate and allowed to form a gel layer at 37 °C. Rat bone marrow-derived mesenchymal stem cells (rBMSCs;PROCELL;CP-R131) were seeded onto the gel surface and cultured for 72 h. The culture medium was removed, and the gels were washed twice with PBS. A staining solution (100 μL) containing Calcein-AM and Propidium Iodide (PI, Solarbio;CA160) was added, followed by incubation in the dark for 30 min. After PBS washing, samples were observed and imaged using an inverted fluorescence microscope (Nikon Eclipse Ti-E) under FITC and TRITC channels.

### 2.6 Preparation of *Staphylococcus aureus* suspension

*Staphylococcus aureus* ATCC 25923 (FENGHU ISHENGWU,China,XJZ037) was streaked onto Tryptic Soy Agar and incubated at 37 °C for 18 h. ATCC 25923 was used as a standardized reference strain in this proof-of-concept study. This strain was selected because it is well characterized, readily available, and exhibits stable growth and biofilm-forming properties, thereby allowing the HAIBF procedure to be established under controlled and reproducible experimental conditions. A single colony was inoculated into 10 mL of Tryptic Soy Broth and incubated at 37 °C with shaking at 200 rpm for 18 h. Five milliliters of the culture was centrifuged at 5000 × *g* for 5 min, and the pellet was washed twice with PBS. The bacteria were resuspended, the concentration was calibrated via plate counting, and the suspension was adjusted to 1 × 10^7^ CFU/mL with PBS and stored at 4°C until use.

### 2.7 Preparation of *Staphylococcus aureus* biofilm fragments

Biofilms were cultured in 24-well polystyrene plates for 48 h at 37 °C in 5% CO_2_ to generate preformed biofilm material. Before preparation of the implantation formulation, total attached biomass was assessed by crystal violet staining, and biofilm morphology was examined by SEM. These assessments verified the presence of preformed biofilm material before fragmentation and implantation. Planktonic bacteria were removed by gentle washing with PBS. Biofilms were scraped from the surface using a cell scraper and fragmented by sonication (30% amplitude, 3 pulses, 30 s total, in an ice-water bath). The resulting fragments were filtered sequentially through 100 μm and 50 μm cell strainers, resuspended in PBS, and adjusted to a final concentration of 2 × 10^7^ CFU/mL.

### 2.8 Effect of PF127 on *S. aureus* biofilm formation

To assess the effect of PF127 on total surface-associated biofilm biomass, *S. aureus* suspensions (1 × 10^7^ CFU/mL) were cultured in tryptic soy broth with or without 15% (w/v) PF127 in 24-well polystyrene plates at 37°C for 1–6 days. Independent wells were used for each time point. After removal of the culture medium, the wells were gently washed twice with sterile PBS and stained with 0.1% crystal violet for 20 min. The bound dye was solubilized with 95% ethanol, and absorbance was measured at 590 nm. PF127-containing wells without bacteria were included as time-matched blank controls. Each condition included 2 technical replicate wells per experiment. Culture medium was replaced every 2 days during incubation. The assay was performed in three independent experiments, and blank-corrected absorbance was used as an indicator of total crystal violet-stained biomass.

### 2.9 Preparation of PF127/Biofilm fragment composite

PF127 powder was dissolved in sterile, ice-cold deionized water under gentle stirring at 4 °C to prepare a 30% (w/v) stock solution. The solution was sterilized by filtration through a sterile 0.22-μm membrane filter and maintained at 4 °C until use. The biofilm fragment suspension prepared as described in section “2.7 Preparation of *Staphylococcus aureus* biofilm fragments” was then mixed with the pre-cooled 30% (w/v) PF127 stock solution at a 1:1 volume ratio. The two components were combined by slow, gentle pipetting at 4 °C until a homogeneous composite suspension was obtained, while avoiding vigorous vortexing or agitation to minimize mechanical disruption of the biofilm fragments. Following 1:1 mixing, the PF127 concentration in the final formulation was 15% (w/v), and the bacterial concentration was adjusted to 1 × 10^7^ CFU/mL. The resulting biofilm fragment–PF127 composite was stored at 4 °C before use. Upon exposure to body temperature, the thermoresponsive formulation underwent a sol-to-gel transition, forming a gel matrix that encapsulated the biofilm fragments.

### 2.10 Animal experiments

#### 2.10.1 Animals and ethics

The experimental protocol was approved by the Institutional Animal Care and Use Committee (IACUC) of Xuzhou Medical University (Approval No. 202411T001) and conducted in accordance with the Guide for the Care and Use of Laboratory Animals.

Forty-eight 8-week-old male SD rats (weight 250–300 g, purchased from Nanjing Laboratory Animal Technology Co., Ltd.) were randomly assigned to four inoculation groups (*n* = 12 per group).

#### 2.10.2 Randomization and blinding

Animals were assigned to the four experimental groups using a computer-generated randomization sequence prepared before surgery by an investigator who was not involved in outcome assessment. Owing to the different appearance and preparation of the inocula, the surgeon performing the inoculation procedure was not blinded to group allocation. However, all specimens were assigned anonymized codes after collection by an independent investigator. Investigators performing micro-CT reconstruction and quantitative analysis, SEM image assessment, CLSM image assessment, histological evaluation, and all quantitative measurements were blinded to group allocation. Group identities were disclosed only after completion of image acquisition, histological scoring/evaluation, quantitative analysis, and statistical analysis.

#### 2.10.3 Grouping and inoculation

Each animal received an injection of 100 μL of the following inoculum into the tibial medullary cavity:

Group A (Planktonic Group): *S. aureus* suspension (1 × 10^7^ CFU/mL).
Group B (Biofilm Group): Biofilm fragment suspension (1 × 10^7^ CFU/mL).
Group C (HAIBF Group): PF127/Biofilm fragment composite suspension (1 × 10^7^ CFU/mL).
Group D (PS Blank Group): Physiological saline.

#### 2.10.4 Surgical procedure

Anesthetized rats were placed in a supine position. A longitudinal skin incision (∼1 cm) was made on the medial side of the left proximal tibia to expose the metaphysis. A bicortical bone defect channel was created using a drill bit (1 mm diameter) at approximately 5 mm below the tibial plateau. One hundred microliters of inoculum was slowly injected into the channel using a micro-syringe to prevent backflow. After implantation, the incision was closed using simple interrupted sutures with 4-0 absorbable polyglactin 910 sutures. The wound edges were carefully approximated, and the knots were placed away from the incision line. The sutures were sufficiently tightened to ensure complete wound closure without causing excessive tissue tension. Animals were housed individually post-operation. The animals were maintained for up to 4 weeks after implantation. Subgroups of rats were euthanized at predetermined time points on postoperative days 7, 14, 21, and 28 for sample collection. Rats were deeply anesthetized with pentobarbital (50 mg/kg, i.p.), then subjected to tibia harvesting. After sampling, an overdose of pentobarbital (150 mg/kg, i.p.) was given, followed by cardiac exsanguination and cervical dislocation. The absence of heartbeat and respiratory movement for more than 2 min was defined as successful euthanasia.

#### 2.10.5 Postoperative monitoring

Animals were monitored daily for general condition, body weight, food and water intake, locomotion, lameness, and wound condition. Supportive care, including softened food and subcutaneous fluid supplementation, was provided when clinically indicated or when progressive body-weight loss was observed. Humane endpoints were applied in accordance with the approved animal protocol.

#### 2.10.6 SEM observation of biofilm formation

For the three inoculated groups (i.e., the planktonic, biofilm, and HAIBF groups), four rats from each group were euthanized on postoperative days 7 and 14, and bone tissue (∼5 × 5 × 2 mm) containing the infection core was harvested. To evaluate biofilm retention and morphology during model establishment, SEM was performed on postoperative days 7 and 14, whereas CLSM was performed on postoperative day 14. Samples were fixed in 2.5% glutaraldehyde for 48 h, washed with PBS, dehydrated through a graded ethanol series, and subjected to critical point drying. After sputter-coating with palladium, samples were observed using a field-emission scanning electron microscope (FEI, USA) at 15 kV. Ten random fields per sample were photographed.

#### 2.10.7 Confocal laser scanning microscopy

On postoperative day 14, tibial specimens containing the inoculation site were harvested from the group of rats in which the osteomyelitis model was established and fixed in 4% paraformaldehyde at 4 °C for 24 h. After washing with PBS, the specimens were decalcified in 10% EDTA (ethylenediaminetetraacetic acid) for 3 weeks and cryoprotected in 30% sucrose solution. The samples were embedded in OCT compound and sectioned longitudinally at a thickness of 8 μm. Sections were incubated with a fluorescently labeled *Staphylococcus aureus*-specific probe (Abcam,AB20920) and counterstained with DAPI to visualize cell nuclei. After washing with PBS, the sections were mounted with an antifade mounting medium and examined using a confocal laser scanning microscope (LSM880). Images were acquired using identical laser power, gain, and exposure settings for all groups to evaluate the distribution and retention of *S. aureus* within the bone defect.

#### 2.10.8 Micro-CT imaging and macroscopic pathological examination

Animals were euthanized on days 21 and 28. Tibiae were harvested and fixed in 4% paraformaldehyde for 48 h. A high-resolution micro-computed tomography (micro-CT) system (Bruker SkyScan 1276, Germany) was used for scanning with the following parameters: 65 kV voltage, 385 μA current, 18 μm resolution, and 1336 rotation steps. A region of interest (ROI) spanning 600 slices (∼2.1 cm) centered on the injection site was selected for three-dimensional reconstruction and quantitative analysis of bone microarchitecture. Macroscopic pathological features such as subcutaneous inflammation and bone necrosis were also documented.

#### 2.10.9 Histological analysis

Tibial bone specimens were decalcified, with the decalcifying solution replaced every 2–3 days until the tissues softened. Following decalcification, the specimens were dehydrated using an automated tissue processor, embedded in paraffin, and sectioned at a thickness of 4 μm. Sections were deparaffinized in xylene, rehydrated through graded ethanol, and stained with hematoxylin for 3–5 min, followed by differentiation, bluing, and eosin counterstaining for 5 min. After dehydration and clearing, sections were mounted with neutral balsam. Images were captured using a light microscope (Nikon Eclipse E100).

### 2.11 Statistical analysis

Data are presented as mean ± standard deviation. Comparisons involving both group and time were performed using two-way ANOVA, followed by Tukey’s multiple-comparisons test. Comparisons among groups at a single time point were performed using one-way ANOVA followed by Tukey’s test. Statistical analyses were conducted using The R Programming Language (version 4.5.3), and *P* < 0.05 was considered statistically significant. Graphs were generated using GraphPad Prism (version 9) and Origin (version 2022).

## 3 Results

### 3.1 Physical characterization and biocompatibility of PF127 hydrogel

First, the thermoresponsive gelation behavior of aqueous PF127 solutions was evaluated using the test-tube inversion method. PF127 solutions at concentrations ranging from 10 to 20% (w/v) exhibited a thermally reversible sol-to-gel transition. At 4°C, all formulations appeared as clear, freely flowing liquids. Their viscosity gradually increased with increasing temperature, and the solutions rapidly transformed into transparent, non-flowing semisolid gels upon reaching a critical gelation temperature (CGT) of 35 ± 2°C (Figures 1A–C). Three representative concentrations (10%, 15%, and 20% [w/v]) were subsequently selected for quantitative characterization of their viscoelastic properties and temperature-dependent gelation behavior using dynamic oscillatory rheometry. During temperature-sweep measurements from 25 to 40 °C, both the storage modulus (G′) and loss modulus (G″) changed markedly. In particular, the 15% (w/v) PF127 formulation exhibited a CGT of 35.1 °C (Figure 1D), indicating that it undergoes gelation at a temperature compatible with the body temperature of rats. Above the transition temperature, G’ exceeded G″ and stabilized at approximately 937.9 Pa, whereas G″ stabilized at approximately 238.5 Pa, confirming the formation of an elastic-dominant semisolid gel.

**FIGURE 1:**
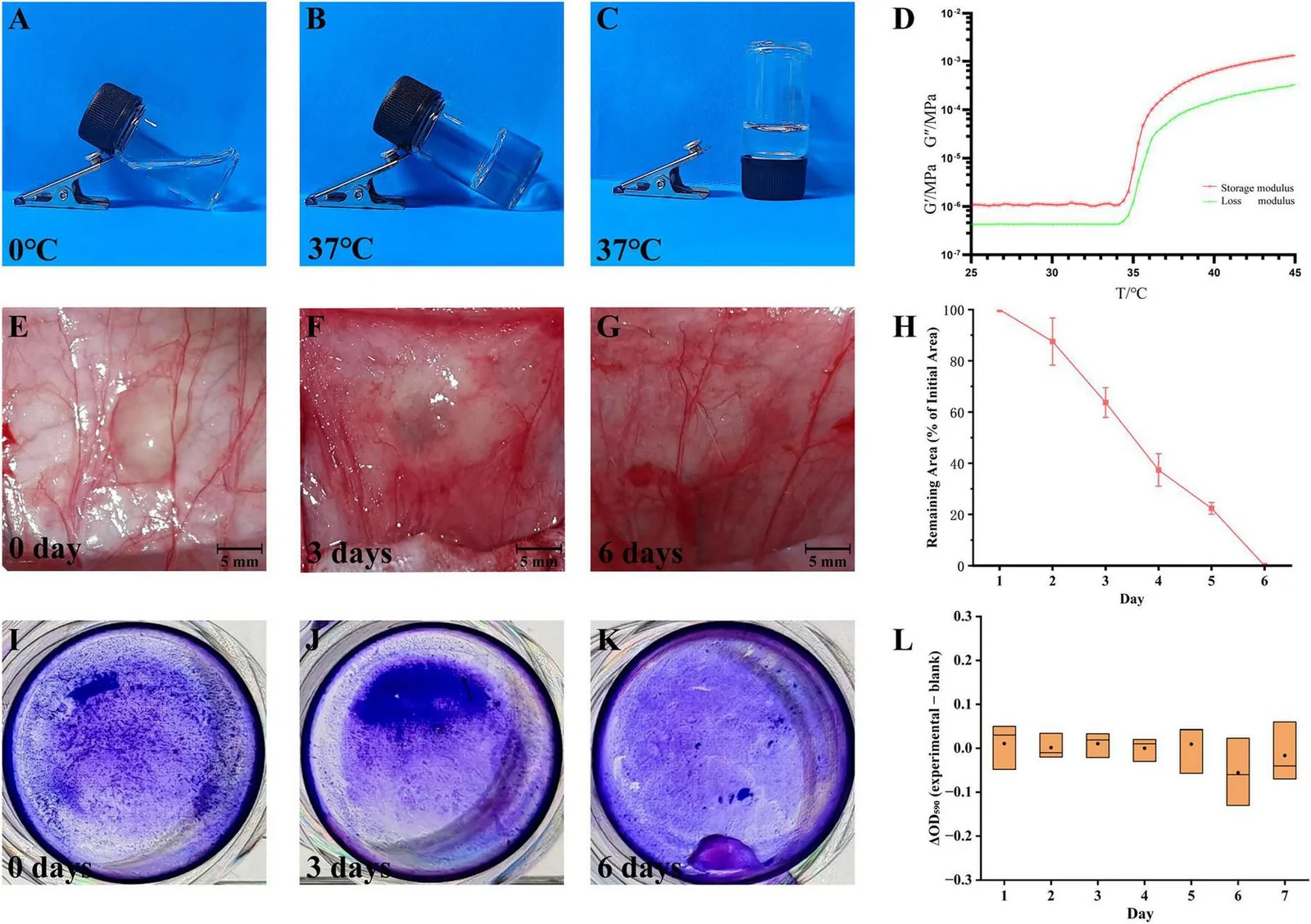
Physical property characterization of PF127. **(A)** PF127 remains in a liquid state at 4 °C. **(B,C)** Upon heating to 37°C, PF127 undergoes a transition to a semi-solid gel. **(D)** Temperature-dependent rheological analysis of 15% PF127. A sharp increase in both the storage modulus (G’, red) and loss modulus (G″, green) is observed at approximately 35.1°C, indicating thermoresponsive *in situ* gelation above this temperature. **(E–G)** *In vivo* degradation following subcutaneous injection of PF127 on days 0, 3, and 6. The long-axis diameter of the injection site measures ∼10 mm initially, shrinks to ∼4 mm on day 3, and is no longer detectable by day 6. **(H)** Quantification of the degradation rate using ImageJ, presented as the percentage change in the degraded area over time. (**I–K)** Assessment of the effect of PF127 on preformed biofilms. Biofilms were pre-cultured for 2 days and subsequently co-incubated with PF127 for 0, 3, and 6 days. Crystal violet staining reveals no apparent disruption of the biofilm structure compared with the untreated control. **(L)** Quantitative analysis of biofilm biomass by measuring the optical density at 590 nm (OD_590_) after crystal violet solubilization, confirming no significant inhibition of biofilm formation by PF127 (*P* > 0.05).

The *in vivo* degradation and resorption of 15% (w/v) PF127 were further evaluated following subcutaneous injection in rats. The initial gel depot had a long-axis diameter of approximately 10 mm, which decreased to approximately 4 mm by day 3 and was no longer macroscopically detectable by day 6 (Figures 1E–G). ImageJ-based quantification of the residual depot area confirmed a progressive reduction over time, with the remaining area expressed as a percentage of the area measured immediately after injection (Figure 1H). No apparent inflammatory response or tissue damage was observed at the injection sites during the observation period.

To assess the effect of PF127 on preformed biofilm biomass, 2-day biofilms were incubated with 15% (w/v) PF127 for 0, 3, or 6 days. Crystal violet staining and absorbance measurements showed no significant change in total stained biomass during the tested period (Figures 1I–K; *P* > 0.05). Biofilm formation was subsequently compared under PF127-containing and PF127-free conditions from days 1 to 6. After correction using PF127-only blanks, both conditions showed comparable temporal changes, with no significant difference in total crystal violet-stained biomass at the tested time points (Figure 1L; all *P* > 0.05). These results indicate that PF127 did not measurably reduce the biomass of preformed biofilms under the tested *in vitro* conditions, supporting its use as a thermoresponsive carrier for model establishment.

Furthermore, the biocompatibility of PF127 was evaluated through bacterial inhibition zone assays and live/dead staining experiments. The bacterial inhibition results showed no clear zone of inhibition surrounding the PF127, in contrast to the significant zone formed around the penicillin-positive control group. This demonstrates that PF127 has no obvious inhibitory activity against *Staphylococcus aureus* (Figures 2A–C). Live/dead staining results revealed that the vast majority of cells co-cultured with the PF127 hydrogel exhibited green fluorescence (calcein-AM labeled, live cells), with only a few cells showing red fluorescence (propidium iodide labeled, dead cells) (Figures 2D, E). Quantitative analysis indicated a cell viability rate exceeding 96%, showing no significant difference compared to the control group (*p* > 0.05). PF127 demonstrated favorable cytocompatibility under the tested *in vitro* conditions, supporting its potential use as a carrier.

**FIGURE 2:**
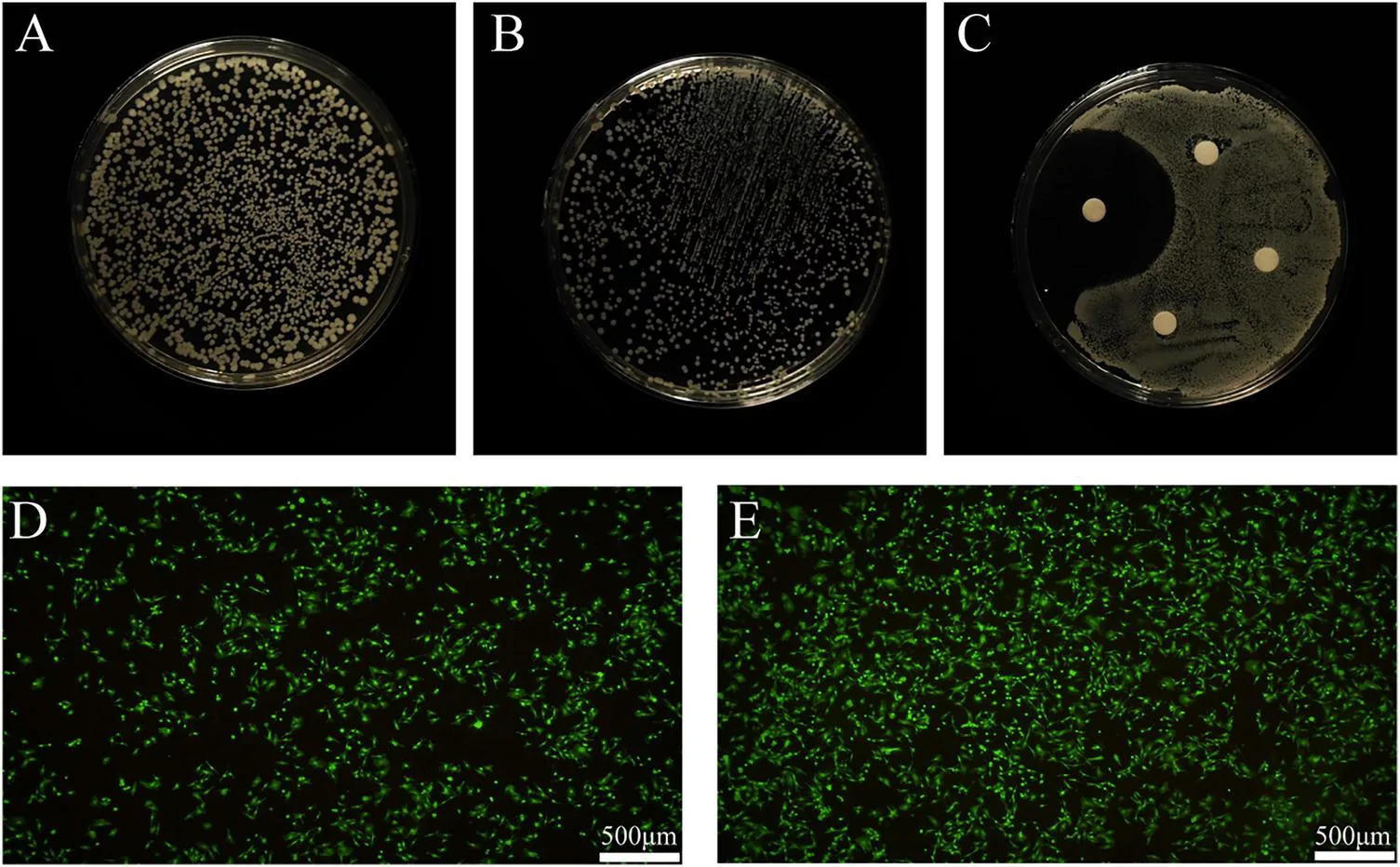
Biocompatibility evaluation of PF127. **(A)** *Staphylococcus aureus* was cultured in TSB to the logarithmic phase (16 h), then inoculated into LB medium and incubated for an additional 24 h (control group). **(B)** *S. aureus* was treated with 15% (w/v) PF127 hydrogel in LB medium for 24 h. The bacteria, immobilized within the hydrogel matrix, were slowly released and proliferated, indicating that their bioactivity was not inhibited. **(C)** Zone of inhibition assay: (1) Penicillin (positive control) produced a zone of inhibition with a diameter of 20 mm, whereas (2) saline (negative control), (3) 15% PF127, and (4) 25% PF127 showed no zone of inhibition. **(D,E)** Fluorescence live/dead staining of rBMSCs co-cultured with PF127 hydrogel for 1 and 3 days. Scale bar, 500 μm. Cell viability was 96%, showing no significant difference compared to the control group (*p* > 0.05). The favorable biosafety profile of 15% PF127 minimizes the confounding effects of the carrier on experimental outcomes.

### 3.2 Animal behavioral observation

Model animals from all groups displayed reduced willingness to move and decreased food intake. Although all animals could move freely and bear weight on the limb of the inoculated side, they exhibited varying degrees of limping. Among all animals that received planktonic injections, only one animal developed a fracture during the observation period. Based on the available clinical findings, the fracture was considered more likely to be pathological than directly related to the experimental procedure, although a definitive cause could not be established. No animal experienced a decrease in body weight exceeding 20% of its preoperative baseline weight during the study period.

### 3.3 Macroscopic pathological findings and mortality

Except for one animal in the planktonic bacteria injection group that died on day 3, probably due to acute infection, all other animals survived until the experimental endpoint (day 28). Local skin temperature in the surgical area of the PS blank group remained stable at 35.0 ± 0.5 °C. Both the planktonic bacteria injection and biofilm inoculation methods showed an increase in local skin temperature at the inoculation site immediately post-surgery, peaking at 37.2 °C. The HAIBF group showed no statistically significant temperature difference compared to the PS blank group by day 4 post-surgery but began to increase thereafter. From day 8, the local skin temperature in the control groups showed a gradual decline, stabilizing at approximately 36.0 ± 0.6 °C after day 14. In contrast, the HAIBF group’s temperature continued to rise before eventually stabilizing at 36.5 ± 0.6°C. Macroscopic pathological results showed that both the planktonic bacteria injection and biofilm inoculation methods led to skin redness, swelling, and subcutaneous abscess formation by day 3, with no obvious signs of infection observed after day 14. Meanwhile, the HAIBF group showed no significant differences compared to the PS blank group within the first 7 days, exhibiting no skin redness or purulent discharge. After that, local skin redness gradually appeared and signs of infection, such as pustules, skin ulceration, and pus discharge, eventually showed up by day 14. These findings suggest that the HAIBF group developed macroscopic features consistent with a more persistent infection course than the planktonic and biofilm-fragment groups, whereas the latter groups showed prominent early inflammatory manifestations.

### 3.4 Confocal and scanning electron microscopy

CLSM and SEM were used to examine the distribution and morphology of bacteria and biofilm-like structures at the inoculation site. In the HAIBF group, CLSM showed more extensive *S. aureus*-associated fluorescence at the bone defect site than in the planktonic-bacteria and biofilm-fragment groups, in which fluorescence was more scattered within the trabecular spaces (Figures 3A–F). SEM further revealed clustered biofilm-like structures in the HAIBF group on postoperative days 7 and 14, whereas predominantly scattered surface-associated structures were observed in the other inoculated groups (Figures 3G–L). Together, these morphological findings are consistent with enhanced local retention and organization of biofilm-derived material in the HAIBF group.

**FIGURE 3:**
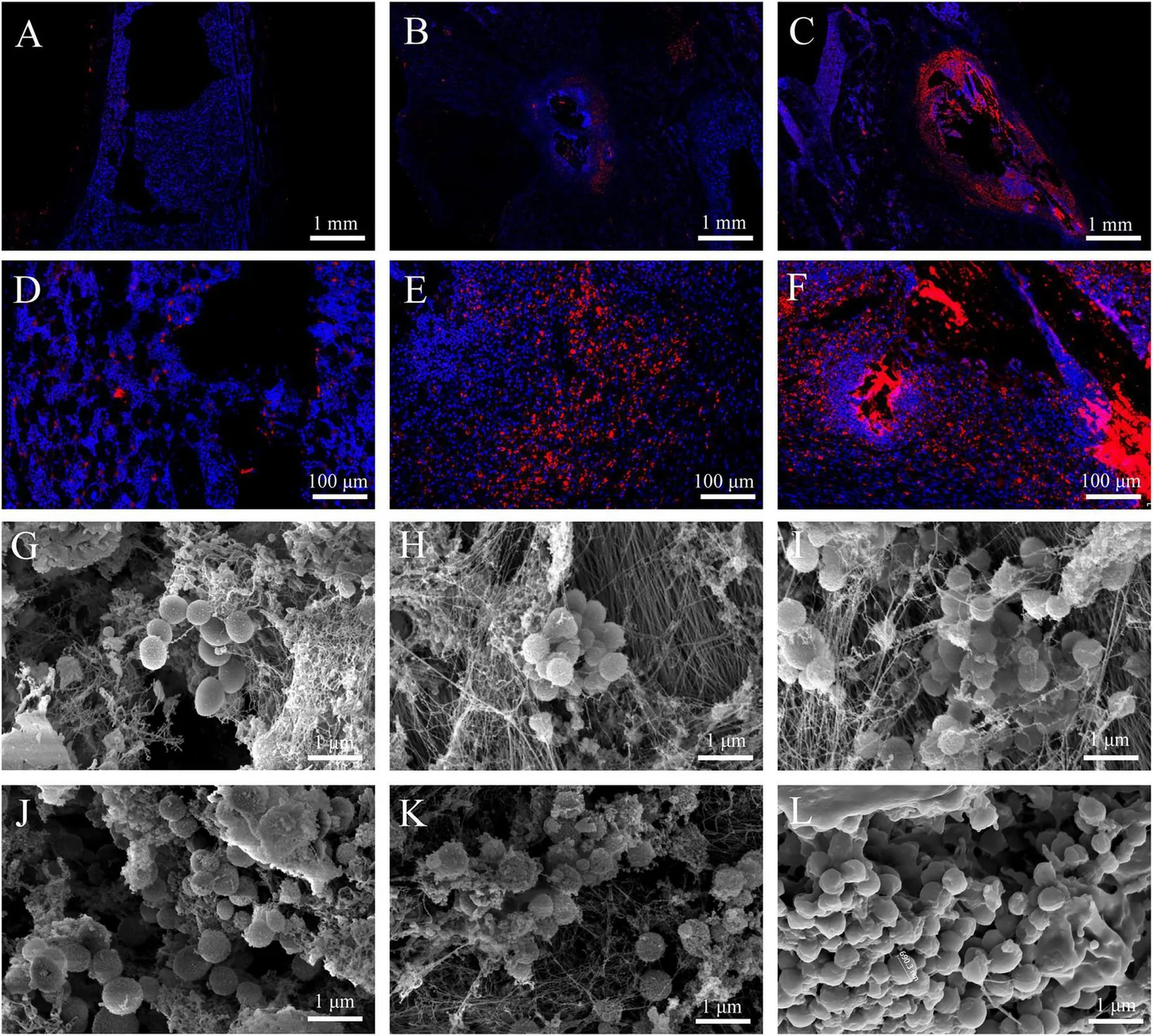
Microscopic characterization of infected bone defects at week 1 and 2 after infection. Confocal laser scanning microscopy (CLSM) images of bone defects at week 2 after infection, stained with DAPI (blue, bone cell nuclei) and a *Staphylococcus aureus*-specific probe (red). **(A)** Planktonic bacteria injection group: sparse and disorganized blue fluorescence (trabecular bone cells) with scattered red signals, indicating poor bacterial colonization and Opotential clearance from the defect site. **(B)** Biofilm fragment injection group: more abundant red fluorescence compared to the planktonic group, suggesting improved bacterial retention. **(C)** HAIBF group: dense and uniformly distributed red fluorescence surrounding the bone defect. Scale bar, 100 μm. **(D–F)** Higher-magnification images of Planktonic bacteria injection group, Biofilm fragment injection group, HAIBF group, respectively. Scale bar, 100 μm. Scanning electron microscopy (SEM) images of *S. aureus* within bone defects at week 1 after infection. **(G)** Planktonic bacteria injection group: scattered bacterial adhesion on bone tissue, with no observable biofilm formation. **(H)** Biofilm fragment injection group: similar to the planktonic group, with no obvious extracellular polymeric substance (EPS) production. **(I)** HAIBF group: conspicuous bacterial aggregates attached to the bone tissue, with individual bacteria exhibiting smooth surfaces. SEM images of *S. aureus* within bone defects at week 2 after infection. **(J)** Planktonic bacteria injection group: bacterial surfaces appear roughened, indicative of host-bacterium interactions. **(K)** Biofilm fragment injection group: poor bacterial aggregation and rough surface morphology. **(L)** HAIBF group: thickened biofilm observed, characterized by multilayered cells embedded within EPS. Scale bar, 1 μm.

### 3.5 Micro-CT scan results

Micro-computed tomography (micro-CT) was used to evaluate bone destruction and new bone formation at 3 and 4 weeks after infection. Three-dimensionally reconstructed images showed that the PS blank group had intact, smooth-surfaced cortical bone with continuous trabecular structure. The planktonic bacteria injection and biofilm inoculation groups exhibited severe osteolytic lesions, characterized by enlarged medullary cavities and extensive trabecular bone loss. In contrast, the HAIBF group displayed disordered and thickened trabeculae (Figures 4, 5). Quantitative analysis revealed that compared to the PS blank group, the planktonic bacteria injection group, biofilm-fragment group, and HAIBF group exhibited significant reductions in trabecular number (Tb.N) by 62.56%, 56.14%, and 42.3% (*p* < 0.01), confirming bacteria-induced bone resorption. Trabecular thickness (Tb.Th) was decreased by 51.92% and 45.91% in the planktonic bacteria and biofilm-fragment groups (*p* < 0.01), whereas the HAIBF group showed a 22.9% increase in Tb.Th. This finding indicates an alteration in trabecular microarchitecture in the HAIBF group; however, micro-CT alone cannot determine whether this change results from altered osteogenic activity, impaired bone resorption, compensatory sclerosis, or other infection-associated remodeling processes. No significant differences were observed between the two time points for most parameters. The only exceptions were Tb.Th in the biofilm group and Tb.N in the PS group, for which significant differences were observed between 3 and 4 weeks. Furthermore, at week 3, the reductions in bone mineral density (BMD) and bone volume fraction (BV/TV) in the HAIBF group were 8.39% and 5.91%, respectively, compared with the PS blank group (Figures 6, 7). These changes were less pronounced than those observed in the planktonic bacteria and biofilm groups, indicating a distinct pattern of infection-associated bone microarchitectural alteration in the HAIBF group. Additionally, particular attention was given to the structural model index (SMI) and connectivity density (Conn.D). In the planktonic bacteria injection group and the biofilm-fragment group, SMI increased while Conn.D decreased (Table 1). In contrast, the HAIBF group exhibited a remarkable reduction in SMI along with a decline in Conn.D, which distinguishes it from simple bone defect healing or acute bone destruction. These findings demonstrate a distinct bone microarchitectural pattern in the HAIBF group compared with the predominantly osteolytic changes in the planktonic-bacteria and biofilm-fragment groups. Its biological interpretation is considered in the Discussion.

**FIGURE 4:**
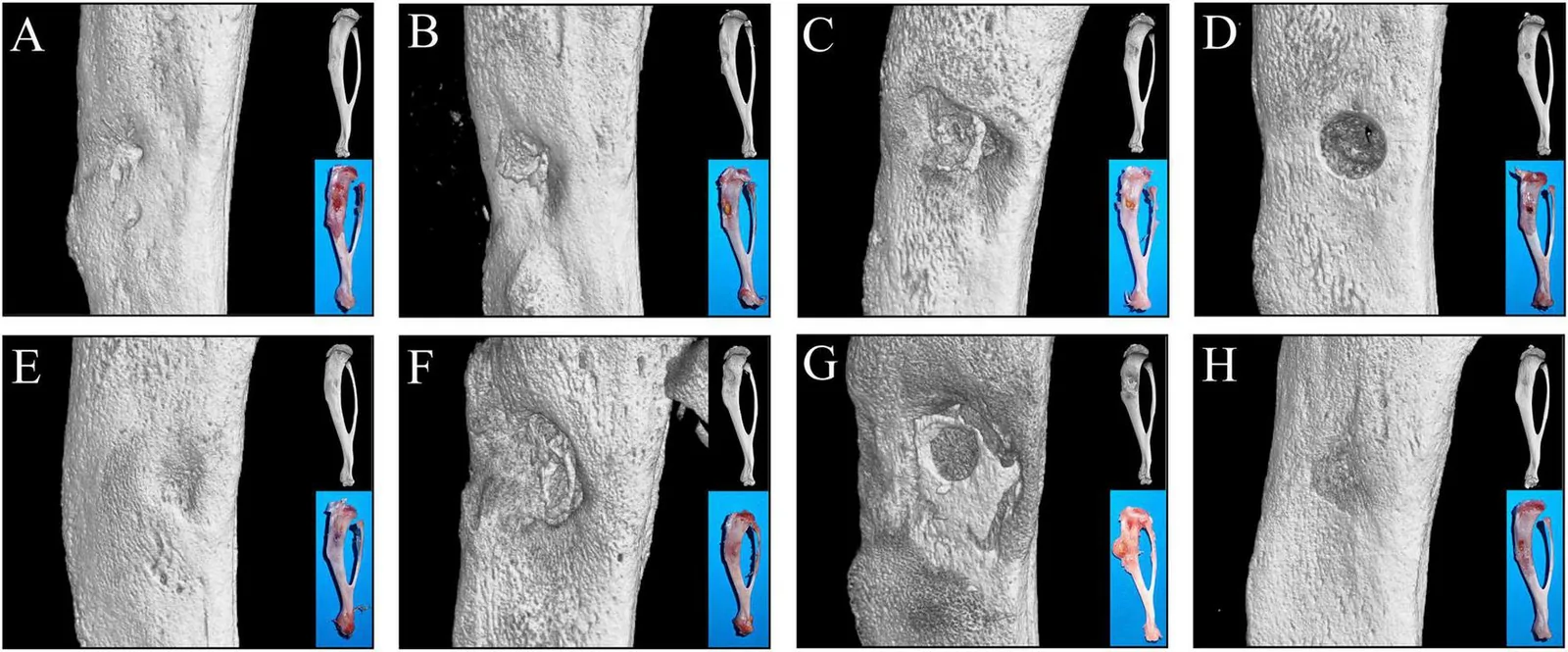
Acroscopic pathological and micro-computed tomography (micro-CT) three- dimensional reconstruction of tibiae. At week 3 after infection. **(A)** Planktonic bacteria injection group: Pus was observed at the bone defect site. Three-dimensional reconstruction revealed bone destruction and trabecular bone loss, presenting the remarkable bone resorption. **(B)** Biofilm fragment injection group: Abundant purulent pus was present at the bone defect. Three-dimensional reconstruction showed no significant difference compared to the planktonic bacteria injection group. **(C)** HAIBF group: Profuse purulent pus was observed at the bone defect. Three-dimensional reconstruction revealed severe bone destruction, accompanied by thickened and disorganized trabecular bone architecture. **(D)** PS injection group: No purulent pus was evident. Three-dimensional reconstruction showed an intact bone structure with a regular circular defect and well-organized trabecular bone, with no signs of bone destruction. At week 4 after infection. **(E)** Planktonic bacteria injection group: No obvious signs of infection were observed. Three-dimensional reconstruction findings were similar to those at week 3, with no evidence of progressive infection. **(F)** Biofilm fragment injection group: No purulent pus was noted, although traces of bone destruction were visible. Three-dimensional reconstruction revealed mildly thickened and disorganized trabecular bone. **(G)** HAIBF group: A distinct purulent mass was present at the bone defect site, indicating persistent infection. Three-dimensional reconstruction showed further bone destruction and pathological bone formation characterized by trabecular thickening and disorganization. **(H)** PS injection group: The regular bone defect was reduced in size, indicative of normal bone healing.

**FIGURE 5:**
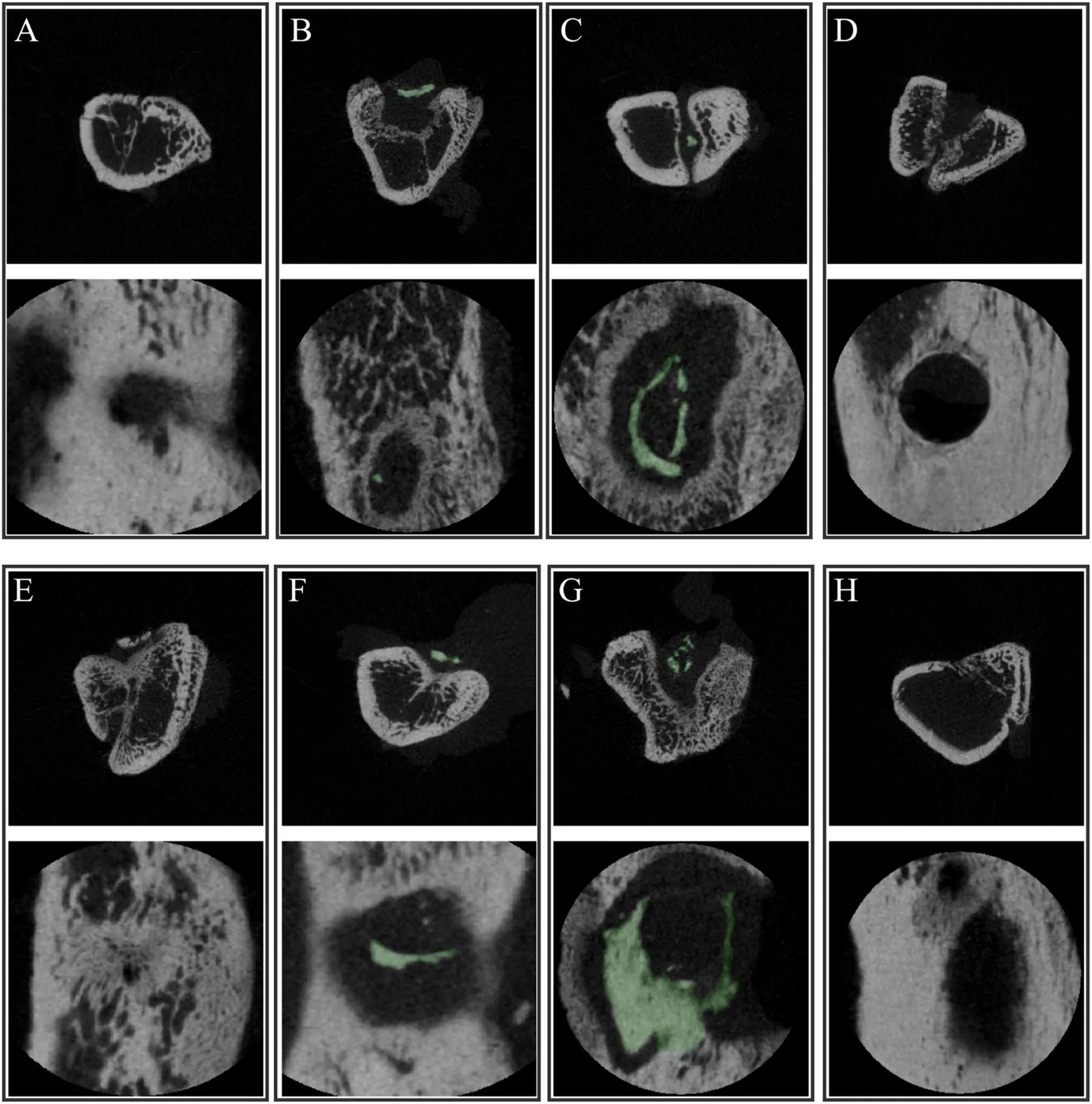
Micro-computed tomography (micro-CT) tomographic images. Magnified views of tibial bone defects. Insets in the upper right corner show the whole tibia, and images in the lower right corner present the corresponding macroscopic pathology. **(A,E)** Planktonic bacteria injection group, showing resolution of infection or a trend toward self-healing. **(B,F)** Biofilm fragment injection group, exhibiting a small amount of sequestrum and sparse trabecular bone structure. **(C,G)** HAIBF group, displaying heterogeneous trabecular bone aggregation and isolated bony encasement. **(D,H)** PS injection group, demonstrating normal medullary bone healing.

**FIGURE 6:**
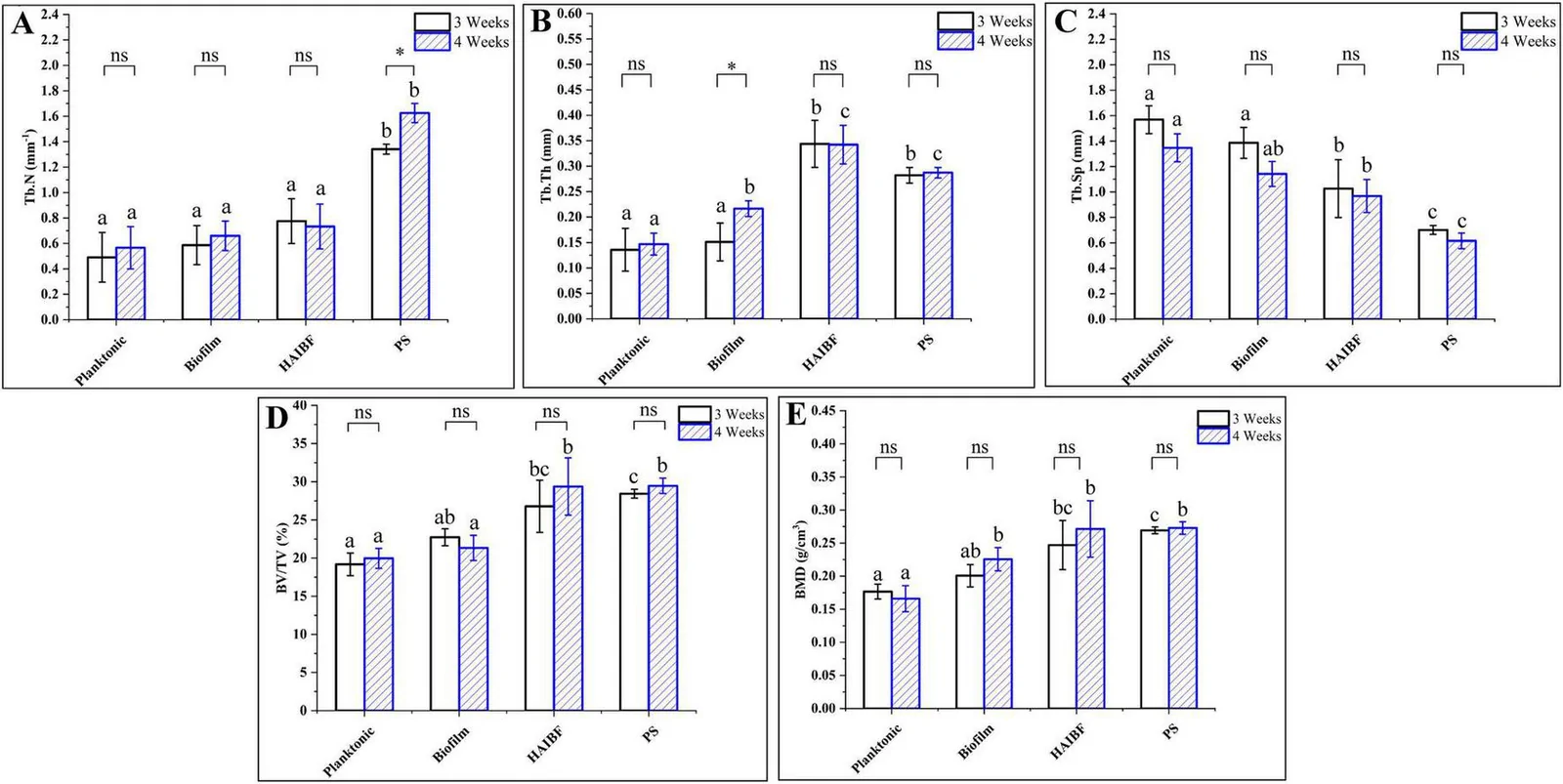
Quantitative micro-CT analysis of the region of interest in bone defects at 3 and 4 weeks after infection. Quantitative parameters of trabecular number, trabecular separation, trabecular thickness, bone mineral density (BMD), and bone volume fraction at three and 4 weeks. **(A)** Trabecular Number (Tb.N): All groups showed a decrease. **(B)** Trabecular Thickness (Tb.Th): The control group decreased, while the HAIBF group showed an increase. **(C)** Trabecular Separation (Tb.Sp): All three groups exhibited an increase. **(D)** Bone Volume Fraction (BV/TV): The mean value for the HAIBF group was close to that of the blank control group. **(E)** Bone Mineral Density (BMD): The mean value for the HAIBF group was close to that of the blank control group. Data are presented as mean ± SD (*n* = 4 per group). Statistical analysis was performed using two-way ANOVA followed by Šídák’s multiple-comparisons test. Different lowercase letters indicate statistically significant differences among groups within the same time point (*P* < 0.05). No significant differences were observed between the two time points for most parameters. The only exceptions were Tb.Th in the biofilm group and Tb.N in the PS group, for which significant differences were observed between 3 and 4 weeks. *p <0.05; **p < 0.01; ***p < 0.001.

**FIGURE 7:**
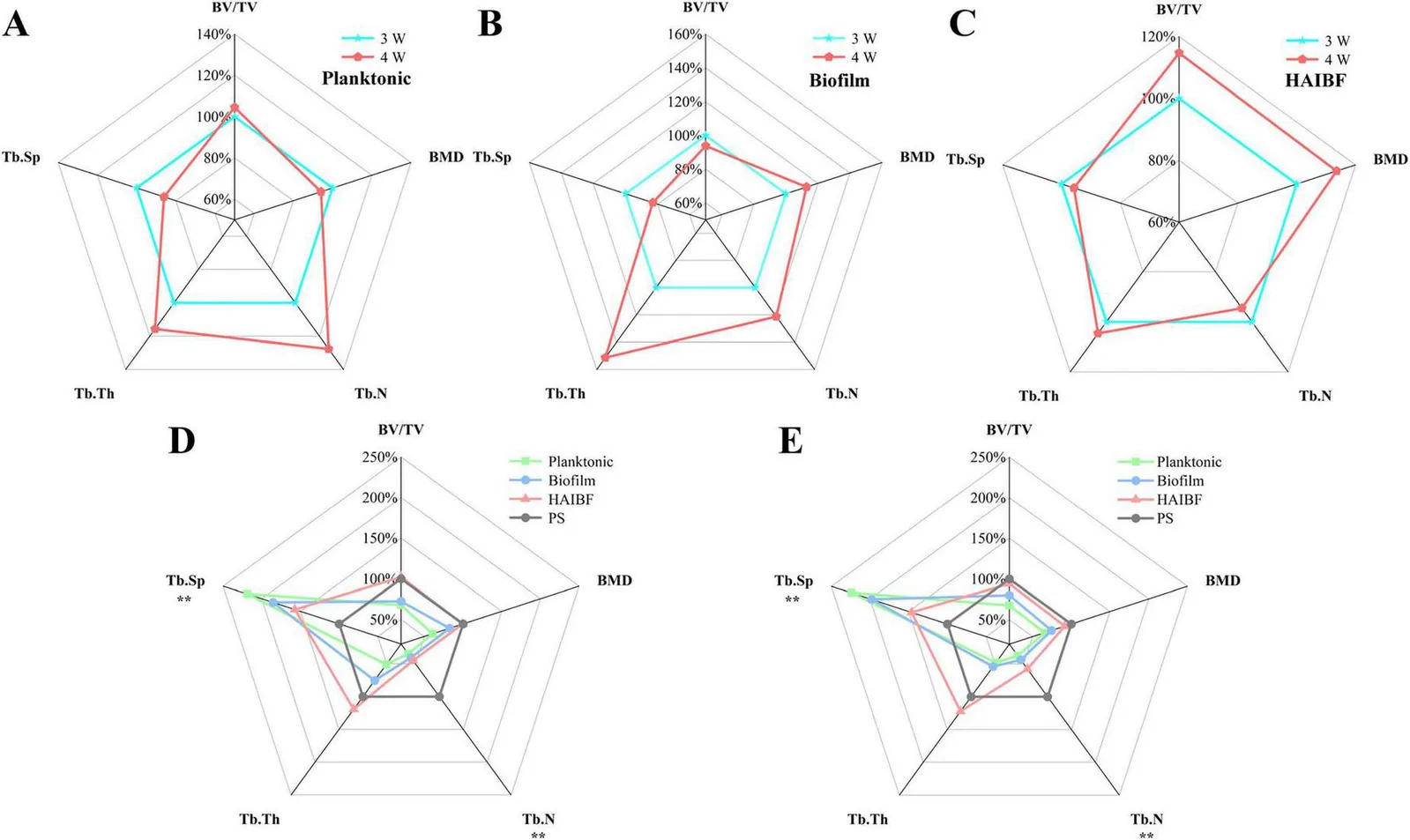
Radar chart integrating five normalized parameters. Trabecular Number (Tb.N), Trabecular Thickness (Tb.Th), Trabecular Separation (Tb.Sp), Bone Volume Fraction (BV/TV), and Bone Mineral Density (BMD). The chart illustrates the trend of changes from week 3 to week 4 for each group (*n* = 4 per group, data points represent group means). **(A)** Planktonic bacteria injection group: An increase in Tb.N and a mitigation of the osteolytic response were observed, suggesting a tendency for the infection process to stagnate. **(B)** Biofilm injection group: An increase in both Tb.Th and Tb.N, along with a further decrease in Tb.Sp, indicated a transition of the infection toward a chronic state. **(C)** HAIBF group: All parameters tended to stabilize, with increases in BMD and BV/TV. This suggests that the rates of bone resorption and pathological bone repair reached a relative equilibrium, leading to the formation of a dense, necrotic bone sequestrum. **(D)** At week 3, compared to the control group, the HAIBF group showed minimal changes in BV/TV and BMD. The trends for Tb.N and Tb.Sp were less than threefold different from the control group. However, Tb.Th exhibited an opposite trend to the control group, showing a significant increase of 22.9%. This indicates that the HAIBF group displayed pathological features of chronic infection, whereas the control group was characterized by an acute infection predominantly driven by bone destruction. **(E)** By week 4, the planktonic bacteria injection group showed signs of infection stagnation. The biofilm injection group displayed upward trends in Tb.Th and BMD, suggesting a possible transition to chronic infection. The HAIBF group exhibited persistent chronic infection. The radar chart visually demonstrates that the infection in the HAIBF group tends to chronicity, indicating that this method can stably replicate the pathological features of chronic infection and improve the reproducibility of the model.

**TABLE 1:** Computed tomography scanning results and macroscopic pathological analysis results.

Computed Tomography scanning results at week 3					Macroscopic pathology assessment				
Group	SMI	Conn.D (mm^–3^)	Osteolysis	Sequesters	Subcutaneous pus	Fracture occurs	Pus in the bone	Healing occurs	Sacrifice occurs
PS Blank	1.35884	32.70902	0	0	0	0	0	–	0
Planktonic	2.02181	6.31048	8	1	8	1	3	2	1
Biofilm	1.82647	9.86543	8	2	8	0	5	2	0
HAIBF	0.27711	10.25667	8	8	2	0	8	0	0

Scanning was performed using a SkyScan 1276 small animal Micro-CT imaging system from Bruker, Germany, with a resolution of 18 μm. The system’s analysis software was used for microstructural parameter analysis of the bone tissue in the target region, with the analysis range kept consistent across all specimens. There were 8 experimental animals in each group, with 0–8 representing the total number of animals exhibiting the corresponding outcome.

### 3.6 H&E staining results

Histological analysis of the bone defect sites was performed using Hematoxylin and Eosin (H&E) staining at 3 or 4 weeks after infection. The PS blank group showed no signs of inflammatory cell infiltration or medullary necrosis. The planktonic bacteria injection and biofilm inoculation groups displayed extensive trabecular bone loss and massive neutrophil aggregation within the medullary cavity. In the HAIBF group, osteoid-like matrix and osteoblast-like cells were observed in areas adjacent to the bone defect, accompanied by localized lymphocyte infiltration and sequestrum encapsulation. These histological findings are consistent with ongoing bone remodeling in a chronic inflammatory environment; Localized lymphocyte infiltration and sequestrum-like structures were also observed in the HAIBF group (Figure 8). These findings are compatible with a chronic infection-associated inflammatory environment and support the presence of selected chronic osteomyelitis-like features within the observation period.

**FIGURE 8:**
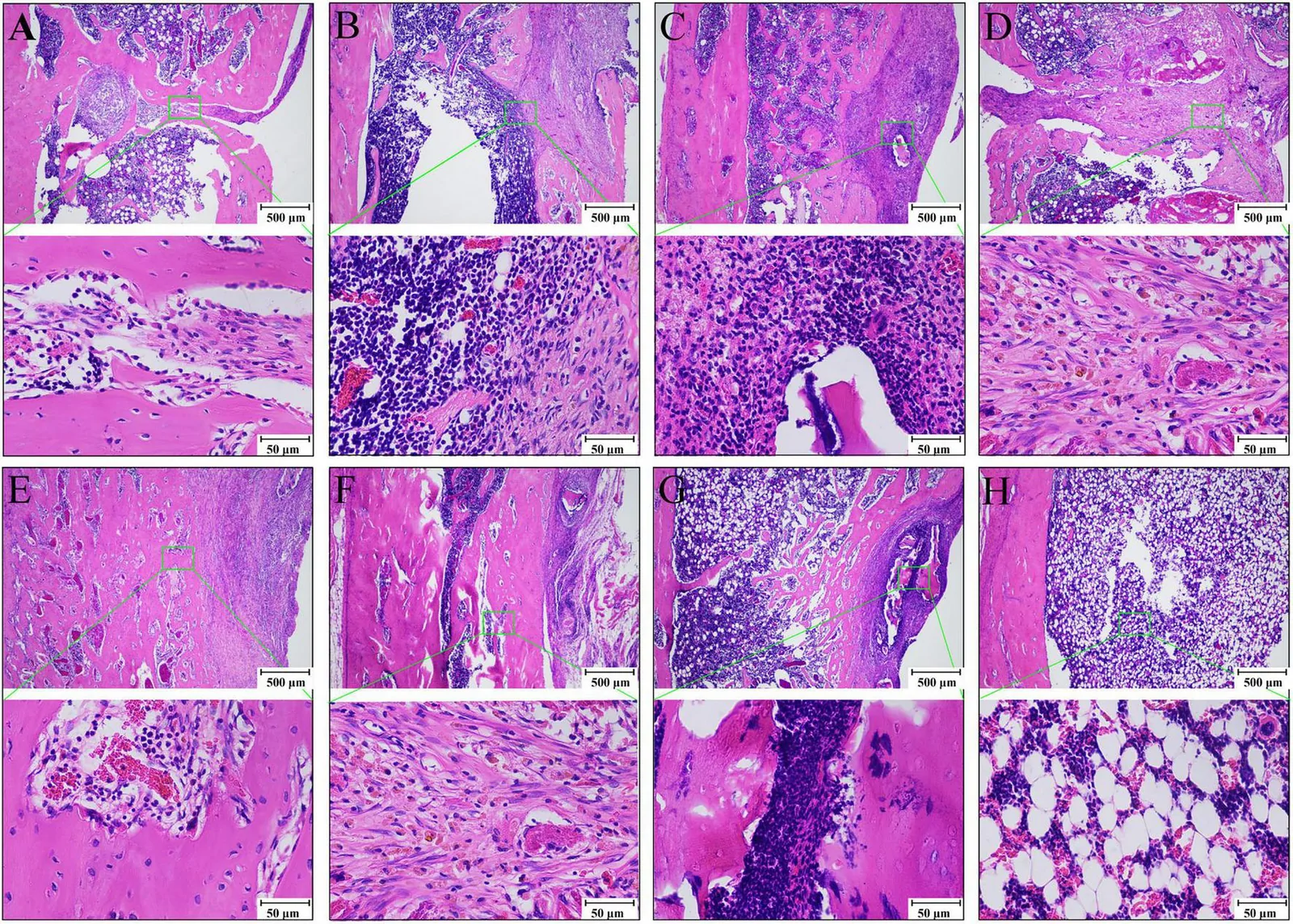
Histopathological analysis of H&E-stained tibial sections at 3 and 4 weeks after infection. The upper panels show representative histological sections of the tibial defect area for each group (Scale bar = 500 μm). The lower panels provide higher-magnification views of the boxed areas, detailing inflammatory infiltration (Scale bar = 50 μm). Staining results at weeks 3 and 4 are shown. **(A,E)** Planktonic bacteria injection group: At week 3, mild neutrophil infiltration was observed. By week 4, inflammatory cells such as neutrophils showed a decreasing trend, indicating resolution of inflammation. **(B,F)** Biofilm injection group: At week 3, the presence of a pathological medullary cavity resulting from destruction, along with extensive neutrophil infiltration, was noted. Findings at week 4 were similar to those at week 3. **(C,G)** HAIBF group: At week 3, bone sequestra and lymphocyte infiltration appeared, indicating the emergence of chronic infection characteristics. At week 4, the pathological features of bone sequestra and lymphocytes persisted. **(D,H)** PS injection group: Normal bone repair was observed, characterized by intact cortical bone, a regular trabecular network, and an absence of inflammatory cells within the medullary cavity.

## 4 Discussion

The curative treatment of chronic osteomyelitis poses a significant clinical challenge, which is due to the high recurrence rates (up to 33%) and prolonged disease course (Leerling et al., 2023; Masters et al., 2019). Therefore, the establishment of reliable animal models is essential for studying its pathological mechanisms and therapeutic strategies (Dao et al., 2023; Ford et al., 2022; Wang X. et al., 2023). Current approaches for establishing implant-associated bone infection models commonly involve inoculation with planktonic bacteria or implantation of biofilm-coated materials. Although planktonic bacterial inoculation is technically simple, the effective bacterial burden and spatial distribution at the target site may vary depending on injection accuracy, local bacterial clearance, tissue dissemination, and host responses (Bernthal et al., 2010; Rodríguez-Merchán et al., 2021; Tande and Patel, 2014). In addition, planktonic inoculation does not necessarily lead to the consistent formation of mature biofilms at the implant site, and the resulting biofilm distribution may be heterogeneous. These factors may limit the reproducibility of chronic implant-associated infection models. Alternatively, implantation of biofilm-coated materials bearing preformed *in vitro* biofilms provides a more direct means of introducing biofilm-associated bacteria. However, the implantation process may affect biofilm integrity and spatial distribution. In addition, the carrier material and implantation procedure may influence the local mechanical environment and host inflammatory response at the bone-implant interface. Achieving consistent control of biofilm biomass, thickness, and spatial coverage can also be challenging. Moreover, an excessively high local bacterial burden may favor an acute inflammatory response or uncontrolled infection rather than a stable chronic infection phenotype (Costerton et al., 1999; Lee and Mooney, 2012; Trombetta et al., 2019). To address these limitations, natural and synthetic hydrogels, including alginate-, gelatin-, chitosan-, hyaluronic acid-, polyethylene glycol (PEG)-, and Pluronic/poloxamer-based systems, have been explored as local carriers for bacterial inoculation or biofilm-associated materials (Burdick and Prestwich, 2011; Li and Mooney, 2016; Zustiak and Leach, 2010). However, these hydrogels differ substantially in their gelation mechanisms and times, degradation kinetics, mechanical properties, swelling behavior, and biological interactions. Therefore, their suitability for the injectable, *in situ* construction of reproducible bone infection models depends on key properties such as injectability, gelation behavior, bacterial compatibility, local retention, degradation profile, and host response (Bodratti and Alexandridis, 2018; Wei et al., 2020). To overcome these limitations, this study aims to construct a more controllable and reproducible animal model of chronic osteomyelitis by optimizing the method of biofilm introduction and regulating its dimensions (Roux et al., 2021).

A novel method, named as HAIBF, involves in-situ coupling thermosensitive hydrogel PF127 with biofilm fragments, in order to simulate the pathological features of chronic osteomyelitis. Specifically, PF127 undergoes a temperature-triggered phase transition into a gel-like semi-solid state upon implantation (Russo and Villa, 2019). This transformation not only immobilizes the biofilm fragments within the target site and facilitates their localized sustained release, but also avoids the potential interference with local bone metabolism associated with the use of inert materials such as bone wax for sealing. Furthermore, the softness and biocompatibility of PF127 can effectively protect and release the conjugated biofilm fragments with a diameter of 50–100 μm, which could overcome irreversible physical damage or structural disruption caused by the size effect in traditional establishment of the infection model methods (Top Hartmann et al., 2024). This is because the fragment diameter exceeds the upper size limit for phagocytosis by macrophages (average diameter ∼20–30 μm) (Krzyżek, 2024; Muthukrishnan et al., 2019; Yang et al., 2024), allowing them to evade immediate clearance by macrophages during the early stages of infection progression, which extends the duration of the local chronic infection process mediated by the biofilm (Høiby et al., 2015).

Multiple characterization methods were employed to validate the significant advantages of HAIBF in terms of establishment of the infection model (Liu et al., 2023; Vanvelk et al., 2018; Zelmer et al., 2022). Overall, scanning electron microscopy (SEM), confocal laser scanning microscopy (CLSM), and macroscopic pathological observation of the models confirmed that PF127 effectively facilitates the colonization and sustained infection of biofilms at the target site. Firstly, SEM observations on days 7 and 14 revealed that the bone defect area in the HAIBF group was covered by a continuous and uniformly distributed biofilm. This biofilm exhibited a three-dimensional framework tightly adhering to the bone surface, consistent with improved local retention and progressive organization of the inoculated biofilm fragments (Peterson et al., 2015). Secondly, CLSM observations showed that, compared to the planktonic bacteria injection and biofilm implantation methods, *Staphylococcus aureus* in the HAIBF group exhibited the most optimal growth trend within the bone defect area and concentrated distribution of biofilm within the trabecular bone structure (Mirzaei et al., 2022). Additionally, a uniformly distributed and well-encapsulated layered biofilm structure was observed on the bone matrix surface. These results indicate that PF127 can facilitate *in situ* retention and organization of inoculated biofilm fragments at the bone defect site, supporting the establishment of a persistent biofilm-associated infection model. In addition, macroscopic pathological results showed that in the early stage of model establishment (within 2 weeks), the HAIBF group did not develop subcutaneous abscesses or acute lethal infections, suggesting that it effectively inhibited bacterial migration to surrounding tissues during this period. In contrast, the other two model establishment methods exhibited significant signs of subcutaneous infection. In the later stage of model establishment (week 4), purulent secretions were still continuously detected at the model establishment site in the HAIBF group, demonstrating the persistent and incurable property of the infection. In the planktonic bacterial injection group and the biofilm suspension injection group, however, there were no obvious signs of infection by week 3, and only residual bone destruction was observed by week 4, suggesting that the infection might have been self-resolved by the host. The above three characterization methods fully illustrate that the PF127 hydrogel can sustain the release of biofilms *in situ* at the bone defect site, overcoming the difficulties of introducing biofilms uniformly and stably compared with traditional methods, and thus better simulating the pathological model of chronic osteomyelitis (Cleaver and Garnett, 2023).

Furthermore, micro-CT and H&E staining were used to evaluate bone structural changes and histopathological features during infection progression. Micro-CT three-dimensional reconstruction and quantitative analysis revealed a distinct pattern of infection-associated bone microarchitectural alteration in the HAIBF model (Zelmer et al., 2022). On one hand, the trabecular number (Tb.N) in all groups showed varying degrees of reduction, confirming that bacterial infection led to definitive bone resorption. However, compared to the methods of injecting planktonic bacteria and implanting biofilms, the HAIBF group exhibited significantly higher trabecular thickness, even exceeding that of the blank group, yet its trabecular separation was relatively low. This phenomenon suggests a possible irregular bone remodeling, which is due to the continued presence of highly active bacteria and ongoing infection. On the other hand, in the HAIBF group, Although BMD and BV/TV in the HAIBF group remained lower than those in the PS blank group at week 3, the reductions were less pronounced than those observed in the planktonic and biofilm-fragment groups. This pattern suggests a distinct infection-associated remodeling response rather than simple preservation of normal bone architecture. These findings indicate that the HAIBF group exhibited a bone microstructural response distinct from the marked osteolytic changes observed in the planktonic bacteria and biofilm groups by week 3. It is worth noting that the HAIBF group showed a reduction in SMI together with a decrease in Conn.D. These findings indicate altered trabecular morphology and reduced structural connectivity. In addition, H&E staining showed that the planktonic bacteria and biofilm groups were characterized predominantly by acute inflammatory cell infiltration at week 3, whereas the HAIBF group exhibited sequestrum formation and chronic inflammatory features, including lymphocyte and plasma cell infiltration. These histological findings support the presence of a chronic infection-associated pathological environment and are consistent with the abnormal bone microarchitectural changes observed by micro-CT. This means that the HAIBF group has simulated the infectious process of chronic osteomyelitis after 3 weeks (Masters et al., 2022). Combining the characterization results from micro-CT and H&E staining, it is fully demonstrated that the HAIBF method can significantly shorten the model establishment period, improve the reproducibility of model establishment, and stably replicate the pathological characteristics of chronic infection.

In summary, the HAIBF method employs PF127 hydrogel as an agent to couple with biofilm fragments, leveraging the softness and biocompatibility of PF127 to achieve *in situ* sustained release and protection of the biofilm. This strategy successfully overcomes the limitations of traditional methods, such as unstable infection, biofilm heterogeneity, and a tendency toward either acute infection or premature resolution. Multiple characterization confirmed that the PF127 not only achieve *in situ* biofilm retention and sustained release, but also replicates the core pathological features of chronic osteomyelitic destruction with pathological repair within three-week period. This work provides a reliable and reproducible animal model for chronic osteomyelitis research.

The HAIBF platform is not intrinsically limited to ATCC 25923 and may be adapted to clinically relevant *S. aureus* strains, including MRSA (Chen et al., 2022), because the establishment of the infection model strategy is based on the local delivery and retention of preformed biofilm fragments within a thermoresponsive PF127 matrix. Despite its clinical relevance, this study has several limitations. Because the model involves implantation of preformed biofilms (Kragh et al., 2023; Maillard and Centeleghe, 2023), early host–pathogen interactions may primarily reflect the recognition and clearance of an established biofilm rather than the sequential processes of initial bacterial adhesion, proliferation, and gradual extracellular matrix formation that occur during natural infection (Al-Madboly et al., 2024; Baertl et al., 2023; Sahli et al., 2022; Sauer et al., 2022; Xu et al., 2020). This design may also accelerate or alter the local immune response, including the early recruitment of inflammatory cells and the subsequent persistence of macrophage-dominated inflammation, thereby affecting the temporal progression of bone destruction and tissue repair. Consequently, the model may not fully reproduce the clinical course of chronic osteomyelitis. In addition, the potential effects of PF127 degradation on the bone microenvironment were not completely excluded. This study focused on the pathological changes and infection outcomes associated with a single strain, ATCC 25923, and did not assess dynamic bacterial changes or osteogenic and inflammatory markers, such as ALP and inflammatory mediators (Zhao et al., 2023). Meanwhile, the translation of findings from animal models to the treatment of human osteomyelitis remains challenging. Further studies are warranted to elucidate the differences between animal models and humans in terms of microbiome composition and function.

## 5 Conclusion

The chronic osteomyelitis driven by biofilms remains a refractory challenge due to its high recurrence rate. This study developed a Hydrogel Assisted In-situ Biofilm Fabrication (HAIBF) method by coupling thermosensitive PF127 hydrogel with biofilm fragments, which stably mimic the pathological process of biofilm-infected chronic osteomyelitis. Leveraging the softness and favorable biocompatibility of PF127, this method facilitates stable bacterial colonization in bone tissue, thereby successfully establishing a chronic osteomyelitis model characterized by short establishment of the infection model duration, high stability, and great reproducibility. This new establishment of the infection model method provides a reliable animal platform for investigating the pathological mechanisms of chronic osteomyelitis in the context of orthopedic infection research and for advancing innovative therapeutic strategies.

## Data Availability

The original contributions presented in this study are included in this article/supplementary material, further inquiries can be directed to the corresponding author.

## References

[B1] Al-Madboly L. Aboulmagd A. El-Salam M. Kushkevych I. El-Morsi R. (2024). Microbial enzymes as powerful natural anti-biofilm candidates. *Microb. Cell. Fact.* 23:343. 10.1186/s12934-024-02610-y 39710670 PMC11664836

[B2] Baertl S. Gens L. Nehrbass D. Sumrall E. Zeiter S. Mannala G. et al. (2023). Staphylococcus aureus from an acute fracture-related infection displays important bacteriological and histopathologic differences from a chronic equivalent in a murine bone infection model. *Clin. Orthop. Relat. Res.* 481, 2044–2060. 10.1097/CORR.0000000000002753 37439643 PMC10499069

[B3] Beard M. Cobb L. Grant C. Varadarajan A. Henry T. Swanson E. et al. (2021). Autoclaving of poloxamer 407 hydrogel and its use as a drug delivery vehicle. *J. Biomed. Mater. Res. B Appl. Biomater.* 109, 338–347. 10.1002/jbm.b.34703 32827204 PMC8221373

[B4] Bernthal N. Stavrakis A. Billi F. Cho J. Kremen T. Simon S. et al. (2010). A mouse model of post-arthroplasty Staphylococcus aureus joint infection to evaluate in vivo the efficacy of antimicrobial implant coatings. *PLoS One* 5:e12580. 10.1371/journal.pone.0012580 20830204 PMC2935351

[B5] Billings C. Anderson D. (2022). Role of animal models to advance research of bacterial osteomyelitis. *Front. Vet. Sci.* 9:879630. 10.3389/fvets.2022.879630 35558882 PMC9087578

[B6] Bodratti A. Alexandridis P. (2018). Formulation of poloxamers for drug delivery. *J. Funct. Biomater.* 9:11. 10.3390/jfb9010011 29346330 PMC5872097

[B7] Burdick J. Prestwich G. (2011). Hyaluronic acid hydrogels for biomedical applications. *Adv. Mater.* 23, H41–H56. 10.1002/adma.201003963 21394792 PMC3730855

[B8] Camilleri-Brennan J. James S. McDaid C. Adamson J. Jones K. O’Carroll G. et al. (2023). A scoping review of the outcome reporting following surgery for chronic osteomyelitis of the lower limb. *Bone Jt Open* 4, 146–157. 10.1302/2633-1462.43.BJO-2022-0109.R1 37051853 PMC10041339

[B9] Chen H. Zhang J. He Y. Lv Z. Liang Z. Chen J. et al. (2022). Exploring the role of Staphylococcus aureus in inflammatory diseases. *Toxins* 14:464. 10.3390/toxins14070464 35878202 PMC9318596

[B10] Cleaver L. Garnett J. (2023). How to study biofilms: Technological advancements in clinical biofilm research. *Front. Cell. Infect. Microbiol.* 13:1335389. 10.3389/fcimb.2023.1335389 38156318 PMC10753778

[B11] Costerton J. Stewart P. Greenberg E. (1999). Bacterial biofilms: A common cause of persistent infections. *Science* 284, 1318–1322. 10.1126/science.284.5418.1318 10334980

[B12] Dao A. O’Donohue A. Vasiljevski E. Bobyn J. Little D. Schindeler A. (2023). Murine models of orthopedic infection featuring Staphylococcus aureus biofilm. *J. Bone Jt Infect.* 8, 81–89. 10.5194/jbji-8-81-2023 37123502 PMC10134754

[B13] Debs P. Boutin R. Smith S. Babic M. Blankenbaker D. Chandra V. et al. (2024). Chronic nonspinal osteomyelitis in adults: Consensus recommendations on percutaneous bone biopsies from the society of academic bone radiologists. *Radiology* 311:e231348. 10.1148/radiol.231348 38625010 PMC11070610

[B14] Eltaher H. Blokpoel Ferreras L. Jalal A. Dixon J. (2022). Direct contact-mediated non-viral gene therapy using thermo-sensitive hydrogel-coated dressings. *Biomater. Adv.* 143:213177. 10.1016/j.bioadv.2022.213177 36371970

[B15] Ford C. Hurford I. Fulbright L. Curry J. Peek C. Spoonmore T. et al. (2022). Loss of Vhl alters trabecular bone loss during S. aureus osteomyelitis in a cell-specific manner. *Front. Cell. Infect. Microbiol.* 12:985467. 10.3389/fcimb.2022.985467 36204648 PMC9530664

[B16] Høiby N. Bjarnsholt T. Moser C. Bassi G. Coenye T. Donelli G. et al. (2015). ESCMID guideline for the diagnosis and treatment of biofilm infections 2014. *Clin. Microbiol. Infect.* 21, S1–S25. 10.1016/j.cmi.2014.10.024 25596784

[B17] Huang C. Tsai K. Weng S. Lin H. Huang H. Wang J. et al. (2016). Chronic osteomyelitis increases long-term mortality risk in the elderly: A nationwide population-based cohort study. *BMC Geriatr.* 16:72. 10.1186/s12877-016-0248-8 27029408 PMC4815108

[B18] Huang S. Wen J. Zhang Y. Bai X. Cui Z. (2023). Choosing the right animal model for osteomyelitis research: Considerations and challenges. *J. Orthop. Translat.* 43, 47–65. 10.1016/j.jot.2023.10.001 38094261 PMC10716383

[B19] Kragh K. Tolker-Nielsen T. Lichtenberg M. (2023). The non-attached biofilm aggregate. *Commun. Biol.* 6:898. 10.1038/s42003-023-05281-4 37658117 PMC10474055

[B20] Krzyżek P. (2024). What is a biofilm? Lessons learned from interactions with immune cells. *Int. J. Mol. Sci.* 25:11684. 10.3390/ijms252111684 39519236 PMC11546875

[B21] Lee K. Mooney D. (2012). Alginate: Properties and biomedical applications. *Prog. Polym. Sci.* 37, 106–126. 10.1016/j.progpolymsci.2011.06.003 22125349 PMC3223967

[B22] Leerling A. Clunie G. Koutrouba E. Dekkers O. Appelman-Dijkstra N. Winter E. (2023). Diagnostic and therapeutic practices in adult chronic nonbacterial osteomyelitis (CNO). *Orphanet J. Rare Dis.* 18:206. 10.1186/s13023-023-02831-1 37480122 PMC10362746

[B23] Li J. Mooney D. (2016). Designing hydrogels for controlled drug delivery. *Nat. Rev. Mater.* 1:16071. 10.1038/natrevmats.2016.71 29657852 PMC5898614

[B24] Li X. Lai L. A. (2024). booming field of large animal model research. *Zool. Res.* 45, 311–313. 10.24272/j.issn.2095-8137.2024.018 38485501 PMC11017075

[B25] Lin Q. Zhu F. Shiye A. Liu R. Wang X. Ye Z. et al. (2024). Antimicrobial effects of thonningianin a (TA)-loaded chitosan nanoparticles encapsulated by a PF-127 hydrogel in diabetic wound healing. *Int. J. Nanomed.* 19, 12835–12850. 10.2147/IJN.S488115 39651354 PMC11624675

[B26] Liu Y. Gao F. Liu S. Xu Z. Zhao X. Yang F. et al. (2025). A smart CO-releasing MnSiO3-based hydrogel enhances diabetic wound healing through NIR-triggered antibacterial, anti-inflammatory, and pro-regenerative mechanisms. *Mater. Today Bio* 34:102084. 10.1016/j.mtbio.2025.102084 40755899 PMC12318298

[B27] Liu Z. Tang W. Liu J. Han Y. Yan Q. Dong Y. et al. (2023). A novel sprayable thermosensitive hydrogel coupled with zinc modified metformin promotes the healing of skin wound. *Bioact. Mater.* 20, 610–626. 10.1016/j.bioactmat.2022.06.008 35846848 PMC9256661

[B28] Maillard J. Centeleghe I. (2023). How biofilm changes our understanding of cleaning and disinfection. *Antimicrob. Resist. Infect. Control.* 12:95. 10.1186/s13756-023-01290-4 37679831 PMC10483709

[B29] Masters E. Ricciardi B. Bentley K. Moriarty T. Schwarz E. Muthukrishnan G. (2022). Skeletal infections: Microbial pathogenesis, immunity and clinical management. *Nat. Rev. Microbiol.* 20, 385–400. 10.1038/s41579-022-00686-0 35169289 PMC8852989

[B30] Masters E. Trombetta R. de Mesy Bentley K. Boyce B. Gill A. Gill S. et al. (2019). Evolving concepts in bone infection: Redefining;biofilm;acute vs. chronic osteomyelitis, the immune proteome and;local antibiotic therapy. *Bone Res.* 7:20. 10.1038/s41413-019-0061-z 31646012 PMC6804538

[B31] Meroni G. Tsikopoulos A. Tsikopoulos K. Allemanno F. Martino P. Soares Filipe J. F. A. (2022). Journey into animal models of human osteomyelitis: A review. *Microorganisms* 10:1135. 10.3390/microorganisms10061135 35744653 PMC9228829

[B32] Mirzaei R. Sabokroo N. Ahmadyousefi Y. Motamedi H. Karampoor S. (2022). Immunometabolism in biofilm infection: Lessons from cancer. *Mol. Med.* 28:10. 10.1186/s10020-022-00435-2 35093033 PMC8800364

[B33] Muthukrishnan G. Masters E. Daiss J. Schwarz E. (2019). Mechanisms of immune evasion and bone tissue colonization that make Staphylococcus aureus the primary pathogen in osteomyelitis. *Curr. Osteoporos. Rep.* 17, 395–404. 10.1007/s11914-019-00548-4 31721069 PMC7344867

[B34] Peng X. Ding C. Zhao Y. Hao M. Liu W. Yang M. et al. (2022). Poloxamer 407 and hyaluronic acid thermosensitive hydrogel-encapsulated ginsenoside Rg3 to promote skin wound healing. *Front. Bioeng. Biotechnol.* 10:831007. 10.3389/fbioe.2022.831007 35866029 PMC9294355

[B35] Percie du Sert N. Hurst V. Ahluwalia A. Alam S. Avey M. T. Baker M. et al. (2020). The ARRIVE guidelines 2.0: Updated guidelines for reporting animal research. *PLoS Biol.* 18:e3000410. 10.1371/journal.pbio.3000410 32663219 PMC7360023

[B36] Peterson B. He Y. Ren Y. Zerdoum A. Libera M. Sharma P. et al. (2015). Viscoelasticity of biofilms and their recalcitrance to mechanical and chemical challenges. *FEMS Microbiol. Rev.* 39, 234–245. 10.1093/femsre/fuu008 25725015 PMC4398279

[B37] Pimentel de Araujo F. Monaco M. Del Grosso M. Pirolo M. Visca P. Pantosti A. (2021). Staphylococcus aureus clones causing osteomyelitis: A literature review (2000-2020). *J. Glob. Antimicrob. Resist.* 26, 29–36. 10.1016/j.jgar.2021.03.030 33965630

[B38] Pincher B. Fenton C. Jeyapalan R. Barlow G. Sharma H. K. A. (2019). systematic review of the single-stage treatment of chronic osteomyelitis. *J. Orthop. Surg. Res.* 14:393. 10.1186/s13018-019-1388-2 31779664 PMC6883574

[B39] Qing X. Dou R. Wang P. Zhou M. Cao C. Zhang H. et al. (2023). Ropivacaine-loaded hydrogels for prolonged relief of chemotherapy-induced peripheral neuropathic pain and potentiated chemotherapy. *J. Nanobiotechnol.* 21:462. 10.1186/s12951-023-02230-5 38041074 PMC10693114

[B40] Ren Y. Liu L. Sun D. Zhang Z. Li M. Lan X. et al. (2023). Epidemiological updates of post-traumatic related limb osteomyelitis in china: A 10 years multicentre cohort study. *Int. J. Surg.* 109, 2721–2731. 10.1097/JS9.0000000000000502 37247014 PMC10498838

[B41] Rodríguez-Merchán E. Davidson D. Liddle A. (2021). Recent strategies to combat infections from biofilm-forming bacteria on orthopaedic implants. *Int. J. Mol. Sci.* 22:10243. 10.3390/ijms221910243 34638591 PMC8549706

[B42] Roux K. Cobb L. Seitz M. Priddy L. (2021). Innovations in osteomyelitis research: A review of animal models. *Animal Model. Exp. Med.* 4, 59–70. 10.1002/ame2.12149 33738438 PMC7954837

[B43] Russo E. Villa C. (2019). Poloxamer Hydrogels for Biomedical Applications. *Pharmaceutics* 11:671. 10.3390/pharmaceutics11120671 31835628 PMC6955690

[B44] Sahli C. Moya S. Lomas J. Gravier-Pelletier C. Briandet R. Hémadi M. (2022). Recent advances in nanotechnology for eradicating bacterial biofilm. *Theranostics* 12, 2383–2405. 10.7150/thno.67296 35265216 PMC8899562

[B45] Sauer K. Stoodley P. Goeres D. Hall-Stoodley L. Burmølle M. Stewart P. et al. (2022). The biofilm life cycle: Expanding the conceptual model of biofilm formation. *Nat. Rev. Microbiol.* 20, 608–620. 10.1038/s41579-022-00767-0 35922483 PMC9841534

[B46] Sayed S. Shekh M. Song J. Sun Q. Dai H. Xue V. et al. (2023). ISX9 loaded thermoresponsive nanoparticles for hair follicle regrowth. *Mater. Today Bio* 23:100849. 10.1016/j.mtbio.2023.100849 38033366 PMC10682119

[B47] Se A. Li L. Yu M. (2025). Poloxamer-based hydrogel with EGCG and rhEGF for diabetic foot ulcer treatment. *J. Mater. Sci. Mater. Med*. 36:65. 10.1007/s10856-025-06917-z 40788421 PMC12339598

[B48] Tande A. Patel R. (2014). Prosthetic joint infection. *Clin. Microbiol. Rev.* 27, 302–345. 10.1128/CMR.00111-13 24696437 PMC3993098

[B49] Tang Y. Zhang L. Sun R. Luo B. Zhou Y. Zhang Y. et al. (2023). Pulmonary delivery of mucus-traversing PF127-modified silk fibroin nanoparticles loading with quercetin for lung cancer therapy. *Asian J. Pharm. Sci.* 18:100833. 10.1016/j.ajps.2023.100833 37635802 PMC10450418

[B50] Top Hartmann K. Lund Nielsen R. Mikkelsen F. Aalbæk B. Lichtenberg M. Holm Jakobsen T. et al. (2024). Bacterial micro-aggregates as inoculum in animal models of implant-associated infections. *Biofilm* 7:100200. 10.1016/j.bioflm.2024.100200 38803605 PMC11128829

[B51] Trombetta R. de Mesy Bentley K. Schwarz E. Kate S. Awad H. A. A. (2019). murine femoral ostectomy model with hardware exchange to assess antibiotic-impregnated spacers for implant-associated osteomyelitis. *Eur. Cell. Mater.* 37, 431–443. 10.22203/eCM.v037a26 31243755

[B52] Vanvelk N. Morgenstern M. Moriarty T. Richards R. Nijs S. Metsemakers W. (2018). Preclinical in vivo models of fracture-related infection: A systematic review and critical appraisal. *Eur. Cell. Mater.* 36, 184–199. 10.22203/eCM.v036a14 30329147

[B53] Wang S. Zhao Y. Breslawec A. Liang T. Deng Z. Kuperman L. et al. (2023). Strategy to combat biofilms: A focus on biofilm dispersal enzymes. *NPJ Biofilms Microbiomes* 9:63. 10.1038/s41522-023-00427-y 37679355 PMC10485009

[B54] Wang X. Zhang M. Zhu T. Wei Q. Liu G. Ding J. (2023). Flourishing antibacterial strategies for osteomyelitis therapy. *Adv. Sci.* 10:e2206154. 10.1002/advs.202206154 36717275 PMC10104653

[B55] Wei S. Ma J. Xu L. Gu X. Ma X. (2020). Biodegradable materials for bone defect repair. *Mil. Med. Res.* 7:54. 10.1186/s40779-020-00280-6 33172503 PMC7653714

[B56] Xu Y. Dhaouadi Y. Stoodley P. Ren D. (2020). Sensing the unreachable: Challenges and opportunities in biofilm detection. *Curr. Opin. Biotechnol.* 64, 79–84. 10.1016/j.copbio.2019.10.009 31766008 PMC7242124

[B57] Yang B. Shu W. Hu J. Wang Z. Wu J. Su J. et al. (2024). Aberrant expression of SLC7A11 impairs the antimicrobial activities of macrophages in Staphylococcus Aureus osteomyelitis in mice. *Int. J. Biol. Sci.* 20, 2555–2575. 10.7150/ijbs.93592 38725861 PMC11077379

[B58] Yang F. Shi Z. Hu Y. Pang Q. Du T. Song B. et al. (2025). Nanohybrid hydrogel with dual functions: Controlled low-temperature photothermal antibacterial activity and promoted regeneration for treating MRSA-infected bone defects. *Adv. Healthc. Mater.* 14:e2500092. 10.1002/adhm.202500092 40045672 PMC12023822

[B59] Zelmer A. Nelson R. Richter K. Atkins G. (2022). Can intracellular Staphylococcus aureus in osteomyelitis be treated using current antibiotics? A systematic review and narrative synthesis. *Bone Res.* 10:53. 10.1038/s41413-022-00227-8 35961964 PMC9374758

[B60] Zhao A. Sun J. Liu Y. (2023). Understanding bacterial biofilms: From definition to treatment strategies. *Front. Cell. Infect. Microbiol.* 13:1137947. 10.3389/fcimb.2023.1137947 37091673 PMC10117668

[B61] Zustiak S. Leach J. (2010). Hydrolytically degradable poly(ethylene glycol) hydrogel scaffolds with tunable degradation and mechanical properties. *Biomacromolecules* 11, 1348–1357. 10.1021/bm100137q 20355705 PMC3050024

